# A Comparative Study of Selected Hospital-Based Health Professionals’ Perceptions of a Learning Organisation in Five South African Districts

**DOI:** 10.3390/healthcare13162058

**Published:** 2025-08-20

**Authors:** Nombulelo Chitha, Ruth Tshabalala, Onke Mnyaka, Mirabel Nanjoh, Ntiyiso Khosa, Thokoe Makola, Kedibone Maake

**Affiliations:** 1 Independent Researcher, Pretoria 0181, South Africa; buli.chitha@gmail.com; 2 Department of Public Health, Walter Sisulu University, Mthatha 5117, South Africa; rtshabalala@wsu.ac.za (R.T.); omnyaka@wsu.ac.za (O.M.); mnanjoh@wsu.ac.za (M.N.); nkhosa@wsu.ac.za (N.K.); tmakola@wsu.ac.za (T.M.)

**Keywords:** learning organisation, public hospitals, pharmacists, dentists, allied health professionals

## Abstract

**Background/Objectives**: The concept of learning organisations, which rely on research-based evidence and emphasise employees’ continuous learning and professional development, is essential for effectively managing change and enhancing the quality of services. To better understand the effects of implementing learning organisation principles in hospital settings, this study aimed to compare the perceptions of selected health professionals regarding learning organisations across five sub-districts in South Africa’s Eastern Cape province. **Methods**: This study employed a quantitative, cross-sectional survey design to collect data from selected health professionals in 9 hospitals in Eastern Cape province. **Results**: Overall, the study showed wide variations in how different healthcare professionals perceived the commitment and communication of department leaders regarding the importance of learning. Key findings showed that 90.9% of dentists agreed that department leaders communicate the importance of learning. In comparison, 68.8% of allied health professionals and 56.5% of pharmacists agreed that department leaders communicated the importance of learning; 21.7% of pharmacists and 25% of allied health professionals disagreed. **Conclusions**: The study has highlighted significant disparities in the perceptions of selected hospital-based health professionals toward introducing a learning organisation in healthcare facilities. This divergence points to the need for tailored approaches in communicating and implementing LO strategies within hospital settings to ensure that all health professionals are equally engaged and that the benefits of continuous learning are fully realised across disciplines.

## 1. Introduction

A growing body of the literature recognises the importance of healthcare facilities either adopting the characteristics of or becoming learning organisations (LOs) in today’s rapidly changing healthcare environment [1,2]. A learning organisation (LO) is described as an organisation with a robust learning culture and structure that fosters learning mindsets and systems, resulting in continuous transformation and innovation [3]. A LO is an institution in which information is created and transferred, and the behaviours of employees are shaped based on the knowledge obtained [3]. A LO recognises that employees, as integral parts of the system, need opportunities to gain new skills and knowledge to utilise in their working environment to maintain organisational effectiveness [4]. The LO concept is built on five principles: personal mastery, mental models, shared vision, team learning, and systems thinking [5]. Implementing these five principles that characterise LO in health facilities will foster a culture in which continuous learning and adaptation are crucial in improving the quality of health and inspiring healthcare employees with the potential for personal and professional growth [2].

In these fast-changing and competitive environments, developing organisational learning as a competence is much needed, so that health systems can cope with these global changes [6]. The capacity of health systems to respond, learn, and adapt has been brought into focus by the COVID-19 global pandemic, which has necessitated the need for rapid adaptation, continuous learning, and innovative solutions to address health system challenges [7]. Organisations must develop learning as a core value and institutionalise a learning process to enhance the adaptive capacity of health services [7]. Implementing a learning culture in healthcare facilities not only holds hope for improving the quality of professional practice, satisfaction, and lifelong learning but also significantly enhances patient care and safety while lowering costs [8]. For instance, researchers have reported attempts by the Iranian health system to transform Iranian public hospitals into LOs [6,9]. The Ministry of Health and Medical Education in the Islamic Republic of Iran has initiated a reform effort to improve its health system by integrating health management training programmes [10]. Additionally, researchers employed the Analytical Hierarchy Process to assess and rank hospitals in Iran according to criteria related to organisational learning, providing valuable insights for future improvements [11,12,13]. In a study conducted in Jordanian hospitals, researchers found the significant impact of practicing the dimensions of LOs in developing human capital [1].

Despite the extensive documentation of experiences on learning organisations, studies on this subject in the healthcare settings remain scarce [8]. Abushohada and Jamali (2022) reported that although the essence of the LO paradigm is easy to understand, research on this subject in healthcare settings is still characterised by a lack of certainty [1]. Somunoglu et al. (2012) analysed the perceptions of a LO application by employees at an oral and dental health centre in Denizli, Turkey, and concluded that there was a weak understanding of the LO concept among the employees [14]. Gagnon et al. (2015) also highlighted that few studies had analysed the effects of a LO in healthcare settings and conducted a study in a health and social services centre in Quebec, Canada, and reported that LOs seemed to positively affect daily nursing work, particularly concerning knowledge transfer, support for nursing practices, and quality of healthcare [8].

As the National Health Insurance (NHI) Act is being implemented within the South African health system [15], continuous professional development and institutional support for health professionals and facilities at all levels will be essential for achieving sustained improvements in health system performance [7,16]. The concept of LOs, drawing on research-based evidence and investing in employees’ continuous learning and professional development, is crucial to positively managing change and the quality of services [17]. It is well established that clinical education and training are integral to developing health facilities into LOs [1,3,6]. Clinical education and training mean that there is a need for facilitators responsible for students’ clinical education to thoroughly understand the education and training guidelines to ensure that future healthcare professionals are well-equipped to contribute to LO [18]. Therefore, to better understand the impact of introducing principles of LOs in hospital settings, this study aimed to compare selected health professionals’ perceptions of a learning organisation in five sub-districts of South Africa’s Eastern Cape province.

## 2. Materials and Methods

### 2.1. Study Design

This study utilised a quantitative, cross-sectional survey design to collect data from selected health professionals in nine hospitals in South Africa. This approach enables comparisons between various groups of health professionals for this study.

### 2.2. Study Setting

This study was undertaken in five districts of South Africa’s Eastern Cape province: Alfred Nzo, Amathole, Buffalo City, Chris Hani, and OR Tambo. The Eastern Cape is home to over 7.2 million people, making it the fourth most populated province in South Africa; it is second biggest in surface area [19]. The chosen district represents a mix of urban, peri-urban, and rural settings [19].

### 2.3. Population and Sampling

The study population consisted of all public hospitals in the five districts of the Eastern Cape Province. The researchers employed a stratified random sampling method to select 9 hospitals and 65 allied health professionals (audiologists, dieticians, occupational therapists, physiotherapists, radiographers, social workers, speech therapists), 23 pharmacists, and 11 dentists in two phases. The sampling approach used in this study was described in detail in a published protocol [20]. The sampled hospitals included All Saints Hospital, Bisho Hospital, Butterworth Hospital, Cecilia Makiwane Memorial Hospital, Dr. Malizo Mpehle Memorial Hospital, Frontier Hospital, Frere Hospital, Madzikane Ka Zulu Memorial Hospital, and Mthatha Regional Hospital.

### 2.4. Data Collection

This study used a structured self-administered questionnaire, and the survey was based on Senge’s framework of five disciplines of a learning organisation: personal mastery, mental models, team learning, shared vision, and systems thinking [20]. The data collection instrument was standardised and validated to ensure reliability. The questionnaire was developed by the research team and reviewed for content validation by the principal investigator (PI), who was an expert in the field. The reliability of the test questions was evaluated using the Statistical Package for Social Sciences (SPSS) version 29.0. through the calculation of Cronbach’s alpha (α), which offers a quantifiable level of agreement on a standardised scale ranging from 0 to 1, with higher values reflecting more significant agreement among items. Our calculated Cronbach’s alpha value was 0.94, which indicated that participants’ responses across the questions were consistent and reliable.

The questionnaire asked questions on demographic characteristics, discussion of learning expectations, learning as a recruitment value, individualised learning plans, and partnership for capacity development in the selected hospitals. Data were collected between 1 June 2022 and 31 December 2022. Health professionals had two options for completing the questionnaire. The research team distributed hard copies of the questionnaires for the health professionals to fill out. The second option involved sending a questionnaire link created on Google Forms. The research team collaborated with the heads of department at the study sites to share the online questionnaire link via platforms such as WhatsApp, email, and others.

### 2.5. Top of Form Data Analysis

Data were captured and coded in Microsoft Excel and analysed using the Statistical Package for Social Sciences (SPSS) version 29.0. Categorical variables were summarised using frequencies and percentages. The distribution of the numerical data was explored through the Shapiro–Wilk test. The result suggested that the numerical data were not normally distributed; thus, the numerical variables were reported using the median and interquartile range (IQR). The Fisher exact test was used to test for significant differences between professional groups. The level of significance was set at 5% (*p*-value ≤ 0.05) for statistical significance.

### 2.6. Top of Form Ethical Considerations and Consent to Participate

The Provincial Health Research Committee of the Eastern Cape Department has granted approval to access the research sites under reference number: EC_202108_011. Ethical clearance was obtained from the Human Research Ethics Committee of the Faculty of Medicine and Health Sciences at Walter Sisulu University, with Protocol Reference No: 072/2021. This study was conducted in accordance with the International Conference on Harmonisation guidelines for good clinical practice in research involving human participants in South Africa and adhered to the four ethical principles of autonomy, beneficence, non-maleficence, and justice. Written informed consent was acquired from all participants, and participation in the study was entirely voluntary.

## 3. Results

The demographic characteristics of the surveyed health professionals are presented in Table 1. The total number of participants surveyed in the nine hospitals was 99, which included 65 allied health professionals, 23 pharmacists, and 11 dentists. The majority (77.6%) were female. Regarding the distribution of participants according to hospital, participants were spread across several hospitals. Frere Hospital contributed the most, at 23.2%, followed by Cecilia Makiwane Hospital, at 22.2%, with Butterworth and Bisho hospitals contributing the least participants, at 3 each. Regarding age, the youngest participant was 21 years old, and the oldest was 61 years old. The median age was 32 years (IQR = 13 years). Most participants were between the ages of 21 and 35, accounting for 63.6% of the participants.

Table 2 summarises health professionals’ perceptions of their hospitals as learning organisations. The study results reveal statistically significant (*p* < 0.05) differences in most of the assessed domains, except for recruitment discussions (*p* = 0.147) and capacity development partnerships (*p* = 0.129). Regarding the importance of learning among health professionals, 68.4% of health professionals agreed or strongly agreed that department leaders effectively communicated this significance, 22.4% disagreed or strongly disagreed, and 9.2% were uncertain. Notably, 90.9% of the dentists agreed or strongly agreed. In comparison, 68.8% of allied health professionals and 56.5% of pharmacists agreed or strongly agreed, while 21.7% of pharmacists and 25% of allied health professionals disagreed or strongly disagreed. A smaller proportion of uncertainty was observed, with 21.7% of pharmacists and 6.3% of allied health professionals reporting uncertainty. Additionally, this disparity in communication perceptions is reflected in the assessment of leadership’s involvement in monitoring learning progress. An impressive 91% of dentists felt leaders actively engaged in this process. In contrast, only 17.4% of pharmacists shared this view, while 52.2% disagreed. Allied health professionals displayed a more balanced perspective, with 40.6% in agreement, 39.1% in disagreement, and 20.3% uncertain, indicating the potential correlation between perceived communication effectiveness and the perceived role of leadership in learning oversight. Finally, the support for diverse learning methods further illustrates the varying experiences among health professionals. About 90.9% of dentists supported the idea that a wide range of learning methods was being used to facilitate health professionals’ learning, compared to 46.9% and 30.4% of allied health professionals and pharmacists who supported the statement.

Among all health professionals, only 53.1% agreed or strongly agreed that department leaders discuss expectations for health professionals’ learning with them, while 24.5% disagreed or strongly disagreed, and 22.4% were unsure. However, significant variations were observed among the various professional groups. Most dentists agreed that department leaders actively discuss learning expectations with health professionals, indicating more favourable perception among this group. However, there is a notable disparity in agreement among other health professions; only 53.1% of allied health professionals and 30.4% of pharmacists shared this perspective, suggesting that communication about learning expectations is less effective. This disparity is further highlighted by the uncertainty expressed by pharmacists, with 34.4% unsure and 34.7% disagreeing or strongly disagreeing, while allied health professionals showed similar uncertainty and disagreement. Additionally, the perceptions of leadership’s role in advising new and less experienced professionals align with these findings. An impressive 90.9% of dentists and 67.2% of allied health professionals believed leaders utilise their experience for guidance, emphasising the positive view of leadership within these groups. However, the lower support from pharmacists, with only 47.8% in agreement and significant disagreement and uncertainty, underscores a potential gap in mentorship and guidance for this profession. Furthermore, when examining the importance of learning during recruitment and onboarding, the differences among the professional groups become even more pronounced. While 63.7% of dentists acknowledged the significance of this practice, only 42.2% of allied health professionals recognised it; in contrast, 47.8% of pharmacists disagreed, while 26.1% were unsure.

In assessing health professionals’ perceptions regarding the application of learning to enhance performance and achieve strategic goals, the results indicate that there was a notable difference in agreement levels. Among all health professionals, 69.1% agreed or strongly agreed that learning is effectively integrated into departmental practices, while 19.6% expressed disagreement and 11.3% remained uncertain. Notably, all dentists surveyed agreed, with 63.6% strongly supporting this point of view. Allied health professionals and pharmacists reported similar opinions, with 69.8% and 52.2%, respectively, believing that learning improves performance and meets strategic goals. However, this agreement does not translate uniformly across all learning and strategic alignment dimensions. When assessing health professionals’ views on aligning their learning with the hospital’s strategic goals, significant variations emerge among different professional groups. Only 21.7% of pharmacists agreed that their learning aligns with hospital goals, with 60.9% remaining unsure. In contrast, 54.5% of dentists and 46.0% of allied health professionals reported a sense of alignment, suggesting that while the application of learning is acknowledged, the connection to strategic goals is less clear for some groups, particularly pharmacists.

Health professionals’ perceptions regarding department leaders’ effectiveness in facilitating individualised learning plans aligned with hospital strategic goals reveal a significant disparity. These findings show distinct differences across professional groups; for instance, 54.6% of dentists agreed or strongly agreed with the leaders’ effectiveness, contrasting with only 29.7% of allied health professionals and 13% of pharmacists who shared similar views. Furthermore, the high percentage of allied health professionals (65.2%) and pharmacists (40.6%) who disagreed indicates a critical gap in leadership support for individualised learning, raising concerns about the overall effectiveness of departmental leadership in fostering alignment with strategic goals. The responses show a parallel trend of uncertainty when evaluating the collaboration between leaders and health professionals to enhance the capacity to achieve the hospital’s goals. Approximately 45.5% of dentists and 40.6% of allied health professionals believed leaders effectively partner with them to develop their capacity, yet a significant portion expressed uncertainty, with 27.3% of both groups unsure. Additionally, the disagreement from 32.8% of allied health professionals and 27.3% of dentists reinforces the disconnect in collaboration efforts. In contrast, 56.5% of pharmacists disagreed or strongly disagreed, highlighting a lack of confidence in leadership collaboration across all groups, particularly among pharmacists, which may further hinder the alignment of learning with strategic objectives.

**Table 2:** Health professionals’ perceptions of a learning organisation (n = 99).

Department leaders communicate the importance of learning by health professionals; n (%).	Health professions	SD	D	N	A	SA	Total	*p*-value
	Pharmacist	2 (8.7)	3 (13.0)	5 (21.7)	8 (34.8)	5 (21.7)	23	0.014 **
	Dentist	0 (0.0)	1 (9.1)	0 (0.0)	4 (36.4)	6 (54.5)	11	
	Allied health professionals	4 (6.3)	12 (18.8)	4 (6.3)	38 (59.4)	6 (9.4)	64	
Learning is applied throughout the department to continuously improve performance and achieve strategic goals; n (%). * One allied health professional did not answer the question.	Health professions	SD	D	N	A	SA	Total	*p*-value
	Pharmacist	1 (4.3)	4 (17.4)	6 (26.1)	10 (43.5)	2 (8.7)	23	0.008 **
	Dentist	0 (0.0)	0 (0.0)	0 (0.0)	4 (36.4)	7 (63.6)	11	
	Allied health professionals	3 (4.8)	11 (17.5)	5 (7.9)	36 (57.1)	8 (12.7)	63 *	
Department leaders monitor the learning progress of health professionals and provide feedback; n (%).	Health professions	SD	D	N	A	SA	Total	*p*-value
	Pharmacist	2 (8.7)	10 (43.5)	7 (30.4)	3 (13.0)	1 (4.3)	23	0.003 **
	Dentist	0(0.0)	0 (0.0)	1 (9.1)	5 (45.5)	5 (45.5)	11	
	Allied health professionals	6 (9.4)	19 (29.7)	13 (20.3)	22 (34.4)	4 (6.3)	64	
Health professionals see how their learning is aligned with the strategic goal of the hospital; n (%). * One allied health professional did not answer the question.	Health professions	SD	D	N	A	SA	Total	*p*-value
	Pharmacist	1 (4.3)	3 (13.0)	14 (60.9)	4 (17.4)	1 (4.3)	23	0.006 **
	Dentist	0 (0.0)	1 (9.1)	4 (36.4)	1 (9.1)	5 (45.5)	11	
	Allied health professionals	6 (9.5)	10 (15.9)	18 (28.6)	25 (39.7)	4 (6.3)	63 *	
A wide range of formal and informal methods are being used to facilitate health professionals’ learning in my department; n (%).	Health professions	SD	D	N	A	SA	Total	*p*-value
	Pharmacist	1 (4.3)	6 (26.1)	9 (39.1)	5 (21.7)	2 (8.7)	23	0.018 **
	Dentist	1 (9.1)	0 (0.0)	0 (0.0)	6 (54.5)	4 (36.4)	11	
	Allied health professionals	5 (7.8)	16 (25.0)	13 (20.3)	25 (39.1)	5 (7.8)	64	
Department leaders use their experience to advise new and less experienced health professionals on how to fulfil the functions of their roles; n (%).	Health professions	SD	D	N	A	SA	Total	*p*-value
	Pharmacist	0 (0.0)	7 (30.4)	5 (21.7)	10 (43.5)	1 (4.3)	23	0.019 **
	Dentist	0 (0.0)	0 (0.0)	1 (9.1)	5 (45.5)	6 (54.5)	11	
	Allied health professionals	3 (4.7)	7 (10.9)	11 (17.2)	31 (48.4)	12 (18.8)	64	
Department leaders discuss expectations for health professionals’ learning with them; n (%).	Health professions	SD	D	N	A	SA	Total	*p*-value
	Pharmacist	1 (4.3)	7 (30.4)	8 (34.4)	5 (21.7)	2 (8.7)	23	0.007 **
	Dentist	0 (0.0)	0 (0.0)	0 (0.0)	5 (45.5)	6 (54.5)	11	
	Allied health professionals	5 (7.8)	11 (17.2)	14 (21.9)	27 (42.2)	7 (10.9)	64	
During the recruitment phase and onboarding of new health professionals, learning is part of the conversation as a value of the hospital; n (%).	Health professions	SD	D	N	A	SA	Total	*p*-value
	Pharmacist	1 (4.3)	11 (47.8)	6 (26.1)	4 (17.4)	1 (4.3)	23	0.147 **
	Dentist	1 (9.1)	1 (9.1)	2 (18.2)	4 (36.4)	3 (27.3)	11	
	Allied	4 (6.3)	17 (26.6)	16 (25.0)	23 (35.9)	4 (6.3)	64	
Department leaders are helping health professionals create an individualised learning plan linked to the hospital’s strategic goal; n (%).	Health professions	SD	D	N	A	SA	Total	*p*-value
	Pharmacist	2 (8.7)	13 (56.5)	5 (21.7)	3 (13.0)	0 (0.0)	23	0.001 **
	Dentist	1 (9.1)	0 (0.0)	4 (36.4)	3 (27.3)	3 (27.3)	11	
	Allied health professionals	9 (14.1)	17 (26.6)	19 (29.7)	19 (29.7)	0 (0.0)	64	
Department leaders are partnering with health professionals to develop their capacity to achieve the hospital’s goal; n (%).	Health professions	SD	D	N	A	SA	Total	*p*-value
	Pharmacist	1 (4.3)	12 (52.2)	5 (21.7)	4 (17.4)	1 (4.3)	23	0.129 **
	Dentist	1 (9.1)	2 (18.2)	3 (27.3)	2 (18.2)	3 (27.3)	11	
	Allied health professionals	6 (9.4)	15 (23.4)	17 (26.6)	22 (34.4)	4 (6.3)	64	

* One allied health professional did not answer the question. ** = 5% level of significance using Fisher’s exact test. SD = strongly disagree, D = disagree, A = agree, SA = strongly agree.

## 4. Discussion

The findings of this study provide valuable insights into the perceptions of selected public hospital-based health professionals (allied health professionals, pharmacists, and dentists) in the five districts of South Africa’s Eastern Cape Province. The significant variations observed among the selected health professionals highlight the need for a more refined understanding of how various healthcare professionals experience and engage with their organisation’s learning culture. The data revealed a significant disparity in how dentists, pharmacists, and allied health professionals perceive leadership communication, the application of learning to improve performance, alignment with strategic goals, the use of learning methods, mentorship, learning expectations, and individualised learning plans.

The study found a significant disparity in health professionals’ perceptions of departmental communication about the importance of learning. Overall, 68.4% agreed that leaders communicated this significance effectively, with dentists showing the highest agreement at 90.9%. This indicates that learning is strongly emphasised and well-communicated in dental departments compared to allied professionals and pharmacists. The findings also suggest a significant disparity in how different professional groups perceive leadership’s role in supporting learning. Dentists demonstrate a strong belief that their leaders actively monitor performance and provide feedback, indicating a culture that prioritises continuous learning. This perception may be due to more structured or visible support systems within their field. In contrast, the significantly lower percentages among pharmacists and allied health professionals imply the potential absence of similar mechanisms, which could result in diminished motivation for ongoing development. The findings regarding the use of diverse learning methods further illustrate the professional groups’ varying perceptions. Dentists again reported overwhelming usage compared to allied health professionals and pharmacists. This variation could reflect differences in training paradigms, professional development opportunities, or institutional resources, supporting the use of varied learning methodologies among health professionals [21,22]. The high agreement among dentists across all three themes suggests a positive feedback loop, indicating that effective communication likely fosters a perception of engaged leadership, which, in turn, facilitates the implementation of diverse learning methods. It was reported in a study that securing the top management’s support and employees’ participation in decision-making was not only important but also promising in creating a learning organisation [14].

The effective communication of learning expectations is fundamental in establishing a robust learning organisation [23]. The significant variations observed across health professions highlight important questions about the effectiveness of communication strategies within the hospital setting. A similar trend was observed when professionals were asked whether department leaders discussed learning expectations with them. Dentists had a more positive perception; in contrast, allied health professionals and pharmacists reported significantly less agreement. With the perceptions of leadership’s role in mentoring and guiding new and less experienced professionals, dentists held the most favourable views, with 90.9% agreeing that leaders effectively utilise their experience to guide others. Furthermore, the importance of learning during recruitment and onboarding also emerged as a significant area of disparity among the different professional groups. This disparity in perceptions may reflect differences in the structure and nature of the roles within the different professions. Dentists, who generally report higher levels of agreement, may experience greater autonomy in their roles or be better represented in leadership structures, which could explain their more favourable perceptions [24]. On the other hand, allied health professionals and pharmacists, who often work in multidisciplinary teams, may experience less clear or consistent communication regarding learning expectations [24]. This disparity suggests that leadership practices regarding mentorship may be unevenly applied or perceived across professional groups. As such, hospital management should prioritise the enhancement of mentorship programmes and leadership training to ensure that leaders at all levels are equipped to support the professional growth of their teams. Ensuring new employees, regardless of their professional background, are introduced to learning resources and opportunities from the outset is essential to building a continuous learning and improvement culture [25].

The emphasis on the importance of leadership and mentorship aligns with the study’s findings, indicating that most health professionals perceive learning to be effectively integrated to enhance performance and meet strategic goals. However, only 21.7% of pharmacists believe their learning aligns with hospital goals, which is notably lower than the perceptions of dentists and allied health professionals. Additionally, most pharmacists have a negative view of collaboration with leaders, contrasting with the more positive perceptions of dentists and allied health professionals regarding their leaders’ abilities. The contrast in these perceptions indicates not only a contrast in leadership practices but also reflects differences in professional roles and expectations of leadership. Effective leadership is pivotal in aligning individual learning plans with broader strategic goals in a learning organisation [26]. However, the findings suggest that departmental leaders may not be equally skilled in meeting the needs of all professional groups. The variation in perceptions of leadership involvement in monitoring learning progress also highlights the need for enhanced leadership training. Leaders should be equipped with the skills to actively engage with their teams and monitor learning progress. This reinforces the need for leadership development that is more aligned with the hospital environment’s diverse professional backgrounds and expectations.

The results of this study emphasise the need for healthcare leaders to tailor their approaches to the specific needs and expectations of different professional groups to foster a more inclusive and equitable learning environment [25,27]. A study found that implementing learning organisation practices can support healthcare professionals in enhancing their skills and knowledge and discovering more effective approaches to their duties [22]. Another study established a significant positive correlation between the dimensions of a learning organisation and nursing staff’s performance [28]. The low levels of confidence in leadership, especially among pharmacists and allied health professionals, point to a need for more robust leadership engagement and assessment to ascertain change management interventions to improve the situation. This requires a commitment to transparency, regular feedback mechanisms, and creating inclusive decision-making processes that involve all professional groups in shaping the hospital’s learning culture [26]. By addressing these leadership gaps, hospitals can move closer to becoming true learning organisations, where every professional feels supported in their development and is actively engaged in achieving the organisation’s broader objectives. Research indicates that fostering a workplace that promotes a learning culture can enable healthcare facilities to deliver effective interventions that enhance their employees’ creative behaviour, leading to improved patient care [28,29,30]. The findings emphasise the importance of fostering a culture of continuous learning and professional development across all health professions within the hospital setting. By prioritising learning, providing clear communication and feedback, and aligning learning initiatives with strategic goals, hospitals can enhance their health professionals’ engagement, performance, and job satisfaction, ultimately leading to improved patient care and organisational outcomes [26,28,29,30].

The variations in perceptions may also be influenced by the unique learning cultures and norms within the various healthcare professions [22]. The educational pathways and training programmes for various healthcare professions can instil different values, priorities, and learning and professional development approaches [21,22]. Dentists, pharmacists, and allied health professionals often undergo distinct educational training experiences, which may shape their expectations and perspectives on the importance of continuous learning. Differences in educational backgrounds, training programmes, and professional development expectations could lead to divergent experiences and perspectives on the learning organisation [21,22]. Underlying organisational policies, resource allocation, and the overall prioritisation of learning and development may differentially impact the various health professions, resulting in observed perception disparities [22]. The level of autonomy and decision-making authority granted to different healthcare professions can also shape their perceptions of the learning organisation culture [24]. Healthcare professionals with less autonomy, like most allied health professionals, may perceive a more top-down approach to learning, which could impact their overall perceptions [24]. By examining these potential factors, healthcare organisations can better understand the drivers behind the differences in perceptions and develop more targeted interventions to create a more equitable and inclusive learning environment for all hospital-based health professionals.

The study relied solely on quantitative survey data, which may not provide a comprehensive understanding of the underlying factors that inform the perceptions of learning organisation culture. Qualitative methods, such as in-depth interviews and focus group discussions, could provide deeper insights into the underlying factors, experiences, and factors that impact healthcare professionals’ views of a learning organisation’s culture [22,31]. Triangulating the survey data with other sources of information, such as document analysis, could enhance the validity of the findings [32]. Adopting a mixed-methods approach could provide a more contextualised understanding of the phenomenon. Additionally, while the uneven group sizes, particularly the limited number of dentists (n = 11), may affect the reliability of subgroup comparisons, our analysis still offers preliminary insights into health professionals’ disparities. To address this, we recommend that future research include a priori power analyses to determine the minimum sample sizes for each profession based on anticipated effect sizes and desired statistical power [33]. In addition, pooling data across sites or employing alternative study designs such as cluster sampling may ensure adequate representation and more reliable subgroup analyses. Despite these limitations, the findings of this study provide valuable insights into the differences in perceptions among the three groups of health professionals. The patterns observed highlight real variations in learning perceptions within the same organisational context. These results serve as a crucial foundation for future, methodologically enhanced studies, informing more targeted interventions and deeper qualitative follow-up.

The most literature focuses on the impact of LO dimensions on healthcare facility performance [2,20,27]. This study aimed to help bridge this gap by comparing the perceptions of different health professional groups within the same hospital setting. Future studies could adopt a more comprehensive and representative sampling approach, ensuring a balanced representation of the various healthcare professions. This would enable more robust comparisons between the perceptions of different healthcare disciplines and identify contextual factors that shape the learning organisation culture across diverse healthcare professionals. Additionally, future research could explore the potential linkages between a learning organisation and other organisational outcomes, such as job satisfaction, employee engagement, quality of care, and patient satisfaction. Establishing such connections could enhance the rationale for investing in implementing a culture of organisational learning within healthcare institutions. Policymakers can utilise the results of this research to inform the development of national healthcare workforce strategies and policies that prioritise the development of a learning organisation culture.

## 5. Conclusions

This study has highlighted significant disparities in the perceptions of selected hospital-based health professionals toward introducing a LO in healthcare facilities. The divergence points to the need for tailored approaches in communicating and implementing LO strategies within hospital settings to ensure that all health professionals are equally engaged and that the benefits of continuous learning are fully realised across disciplines. Understanding the influence of organisational learning and leadership on the perspectives of hospital-based health professionals is also crucial for healthcare organisations to devise specific strategies to foster a more united and inclusive learning environment. This involves harmonising leadership approaches, nurturing a culture emphasising learning, ensuring equitable access to learning resources, and fostering transparent communication and collaboration among diverse health professions. Thus, healthcare facilities can maximise the benefits of LO, leading to improved performance, enhanced patient care, and the achievement of strategic goals.

## Figures and Tables

**Table 1:** Demographic characteristics of health professionals.

Variables	Categories	Frequency n = 99	Percentage (100%)
Sex #; n (%)	Female	76	(77.6)
	Male	22	(22.4)
Health Profession; n (%)	Dentist	11	(11.1)
	Pharmacist	23	(23.2)
	Allied Health Professional *	65	(65.7)
Hospital; n (%)	Frere Hospital	23	(23.2)
	Cecilia Makiwane Hospital	22	(22.2)
	Mthatha Regional Hospital	18	(18.2)
	Frontier Hospital	12	(12.1)
	Madzikane Ka Zulu Hospital	7	(7.1)
	Dr. Malizo Mpehle Hospital	7	(7.19)
	All Saints Hospital	4	(4.0)
	Butterworth Hospital	3	(3.0)
	Bisho Hospital	3	(3.0)
Age Groups; n (%)	21–35 years	63	(63.6)
	36–45 years	21	(21.2)
	46–55 years	10	(10.1)
	56–65 years	5	(5.1)
Age, years, Median (IQR)		32 (13)	
	Minimum	21	
	Maximum	62	

# one pharmacist did not report their sex. * audiologists, dietitians, occupational therapists, physiotherapists, radiographers, social workers, speech therapists.

## Data Availability

The raw data supporting the conclusions of this article will be made available by the authors on request.

## Abbreviations

The following abbreviations are used in this manuscript:

LO	Learning Organisation
NHI	National Health Insurance
SPSS	Statistical Package for Social Sciences
IQR	Interquartile range
